# [Retracted] MicroRNA‑509 acts as a tumor suppressor in tongue squamous cell carcinoma by targeting epidermal growth factor receptor

**DOI:** 10.3892/mmr.2024.13176

**Published:** 2024-01-30

**Authors:** Chao Hou, Yan Dong, Fenghe Zhang, Bo Du

Mol Med Rep 16: 7245–7252, 2017; DOI: 10.3892/mmr.2017.7531

Following the publication of this paper, it was drawn to the Editor's attention by a concerned reader that certain of the Transwell cell invasion assay data shown in Fig. 2C on p. 7248 and Fig. 3G on p. 7249 were strikingly similar to data in different form in other articles written by different authors at different research institutes, which had either already been published (some of which have now been retracted), or had been submitted for publication at around the same time.

Owing to the fact that certain of the data in the above article had already been published prior to its submission to *Molecular Medicine Reports*, the Editor has decided that this paper should be retracted from the Journal. The authors were asked for an explanation to account for these concerns, but the Editorial Office did not receive a satisfactory reply. The Editor apologizes to the readership for any inconvenience caused.

